# Clinical utility of aqueous humor polymerase chain reaction and serologic testing for suspected infectious uveitis: a single-center retrospective study in South Korea

**DOI:** 10.1186/s12886-020-01513-x

**Published:** 2020-06-19

**Authors:** Wungrak Choi, Hyun Goo Kang, Eun Young Choi, Sung Soo Kim, Chan Yun Kim, Hyoung Jun Koh, Sung Chul Lee, Min Kim

**Affiliations:** Institute of Vision Research, Department of Ophthalmology, Yonsei University College of Medicine, 211 Eonju-ro, Gangnam-gu, Seoul, 135-720 South Korea

**Keywords:** Aqueous humor, Infectious uveitis, Plasma, Serologic tests, Polymerase chain reaction, Uveitis

## Abstract

**Background:**

This study aimed to assess and compare the clinical value of aqueous humor polymerase chain reaction (PCR) and serologic tests in patients diagnosed with suspected infectious uveitis.

**Methods:**

In this retrospective observational study, data of 358 patients who were diagnosed with suspected infectious uveitis and who underwent aqueous humor PCR testing were analyzed. PCR and serologic test results were compared with the clinical features.

**Results:**

The rates of initial diagnoses for infectious uveitis were higher with PCR (99 patients, 28%) compared to those with serologic tests (38 pateints, 11%). The diagnostic positivity of PCR was 29% for anterior uveitis, 0% for intermediate uveitis, 5% for posterior uveitis, and 30% for panuveitis. In particular, PCR was useful in confirming the diagnosis of cytomegalovirus and varicella-zoster virus infections and *Toxoplasma gondii*-associated uveitis. For PCR test, the sensitivity was 0.431, specificity was 0.985, and the negative and positive predictive values were 0.506 and 0.980, respectively. For IgM test, the sensitivity was 0.151, specificity was 0.970, and the negative and positive predictive values were 0.403 and 0.895, respectively.

**Conclusion:**

Aqueous humor PCR can be a valuable diagnostic tool for confirming the infectious etiology in patients clinically diagnosed with uveitis. PCR had good predictive and diagnostic value for anterior uveitis and panuveitis compared with that for intermediate and posterior uveitis.

## Background

Uveitis, an important cause of visual impairment in developed countries, affects approximately 200 per 100,000 individuals and accounts for up to 10–35% of severe visual impairment cases [1, 2]. Infectious uveitis comprises approximately 10–20% of all uveitis cases [3, 4]. The common pathogens implicated in infectious uveitis are cytomegalovirus (CMV), herpes simplex virus (HSV) types 1 and 2, varicella-zoster virus (VZV), and *Toxoplasma gondii* [5–7]. Thus, early detection of the causative pathogen and appropriate antimicrobial therapy can prevent visual impairment [1].

Several diagnostic tools such as serologic tests, electron or light microscopy, immunoblots, cell cultures, enzyme-linked immunosorbent assay, and Goldmann-Witmer coefficient are available; however, the initial diagnosis of infectious uveitis is mainly based on clinical features alone. Occasionally, such diagnoses can be quite challenging because not all patients present with pathognomonic clinical features of uveitis. Moreover, a miotic pupil or media opacity can mask the pathognomonic features, or an overlap in the phenotypic expression caused by different pathogens can limit the diagnostic capability based on clinical examination alone [8]. Incorrect diagnoses could delay the administration of targeted treatment, thereby resulting in visual impairment, use of drugs with undesirable side effects, and the occurrence of uveitis-related complications [9].

Polymerase chain reaction (PCR) is a fast and reliable method, which can identify common pathogens causing uveitis [10]. Aqueous humor PCR can precisely detect small quantities of pathogenic DNA or RNA [11, 12]. The usefulness of PCR in diagnosing infectious uveitis has been established; however, only a few studies have compared the results of PCR with those of plasma serologic tests. This study aimed to assess and compare the clinical value of aqueous humor PCR and serologic tests in patients diagnosed with suspected infectious uveitis.

## Methods

### Patient enrollment and study design

In this retrospective, observational, single-center study, a systematic evaluation of electronic medical records of all enrolled patients was performed. Patients who underwent aqueous humor PCR for clinically diagnosed infectious uveitis between August 2005 and March 2017 at Yonsei University Health System were enrolled in the study. Data collection included the patients’ medical history, results of complete ocular examinations (visual acuity, intraocular pressure, and fundus examination), and PCR and serologic test results.

The initial diagnosis was based on clinical presentations and outcomes. Aqueous humor PCR and serologic tests were subsequently performed at the discretion of treating physicians.

### Study groups and serologic tests

The medical records of 358 patients who underwent an aqueous humor PCR test for clinically suspected infectious uveitis were retrospectively reviewed. All patients were divided into the following four groups based on the anatomic location of uveitis: anterior, intermediate, posterior, or panuveitis [13, 14]. The results of aqueous humor PCR and serologic tests were compared, and the positivity of each method was analyzed. Common pathogens such as CMV, HSV, VZV, Epstein-Barr virus (EBV), and *T. gondii* were included in the analysis. IgM serologic tests for HSV, VZV, and EBV were performed using the LIAISON® XL Analyzer (DiaSorin S.p.A., Italy) with commercially available kits (LIAISON® HSV-1/2 IgM, LIAISON® VZV IgM, LIAISON® EBV IgM). IgM serologic tests for CMV and *T. gondii* were performed using Vidas (BioMérieux, Lyon, France) with commercially available kits (VIDAS CMV IgM-bioMérieux, VIDAS Toxo IgM-bioMérieux).

### Aqueous humor sampling

Aqueous fluid sampling was performed during slit lamp examination with topical anesthesia under sterile conditions. A 30-gauge needle was used to extract 0.05–0.1 mL aqueous humor. The occurrence of aqueous humor sampling-related complications such as intraocular pressure fluctuation and cataract progression was recorded.

### PCR

Real-time PCR was performed within 24 h of aqueous humor collection according to the manufacturer’s instructions. Samples were analyzed for CMV, VZV, HSV, EBV, or *T. gondii* DNA based on clinical suspicion. The PCR detection threshold (viral copies/mL) was 126 for CMV, 100 for VZV, 154 for HSV, 510 for EBV, and 100 for *T. gondii*. LightCycler 480 (Roche, Forrenstrasse, Switzerland) was used for real-time PCR. Typically, EBV RQ-PCR was performed using the MagNA Pure 24 System (Roche Diagnostics, Forrenstrasse, Switzerland), Rotor-Gene Q (QIAGEN GmbH, Germany), MagNA Pure 24 Total NA Isolation kit (Roche Diagnostics), and Artus® EBV RG PCR kit (Artus GmbH, Hamburg, Germany). For CMV RQ-PCR, CMV Quantification Real-time PCR kit (BioCore, Seoul Korea) was used. Samples for PCR analysis of HSV, VZV, and *T. gondii* were outsourced to Seoul Clinic Laboratories (Seoul, Korea).

### Statistics

Data were analyzed using SPSS 22.0 software (IBM Corp., Armonk, NY, USA). Differences among groups were examined using independent t-test or Wilcoxon signed-rank test. *P*-values < 0.05 were considered statistically significant.

## Results

Of the 358 patients included in this study, 34 had anterior uveitis, 10 had intermediate uveitis, 22 had posterior uveitis, and 292 had panuveitis. The mean age of the participants was 54.05 ± 16.58 (range, 13–84) years. The overall diagnostic positivity of PCR and IgM serologic tests for infectious uveitis was 28% (99/358 patients) and 11% (38/358 patients), respectively (Table 1). The pathogens identified by PCR were 3 cases of HSV, 17 of VZV, 67 of CMV, 2 of EBV, and 10 of *T. gondii*. IgM serologic tests identified 13 cases of HSV, 12 of VZV, 7 of CMV, 4 of EBV, and 2 of *T. gondii* (Table 1).

**Table 1:** Results of polymerase chain reaction and serologic tests in patients with uveitis

	Pathogen	Anatomical classification of uveitis				
		Anterior uveitis (*N* = 34)	Intermediate uveitis (*N* = 10)	Posterior uveitis (*N* = 22)	Panuveitis (*N* = 292)	Total (*N* = 358)
**PCR**	HSV	2	0	0	1	3
	VZV	1	0	0	16	17
	CMV	7	0	1	59	67
	EBV	0	0	0	2	2
	*T. gondii*	0	0	0	10	10
	**Diagnostic positivity**	10/34 (29%)	0/10 (0%)	1/22 (5%)	88/292 (30%)	99/358 (28%)
**Serum IgM**	HSV	1	1	0	11	13
	VZV	1	0	0	11	12
	CMV	0	0	0	7	7
	EBV	0	0	0	4	4
	*T. gondii*	0	0	0	2	2
	**Diagnostic positivity**	2/34 (6%)	1/10 (10%)	0/22 (0%)	35/292 (12%)	38/358 (11%)

Results are presented as *N* or *N* (%); *PCR* polymerase chain reaction, *IgM* immunoglobulin M, *HSV* herpes simplex virus, *VZV* varicella-zoster virus, *CMV* cytomegalovirus, *EBV* Epstein-Barr virus, *T. gondii Toxoplasma gondii*

Ophthalmic findings of HSV-1/HSV-2- and VZV infection included elevated intraocular pressure associated with acute iritis, stellate keratic precipitates throughout the corneal endothelium, large granulomatous keratic precipitates, sectoral or non-sectoral iris transillumination defects, iris pigment epithelium atrophy, decreased corneal sensation, and mydriatic or corectopic pupil at rest. Ophthalmic findings of CMV infection included unilateral (occasionally bilateral) anterior chamber inflammation associated with iris sectoral defects, episodes of ocular hypertension, and diffuse linear or coin-shaped keratic precipitates, occasionally with focal endotheliitis. Ophthalmic findings of *Toxoplasma* infection were partial- or full-thickness necrotizing retinitis adjacent to an old hyperpigmented chorioretinal scar associated with focal arteritis, overlying vitritis, and anterior chamber cells [15, 16].

The sensitivity, specificity, and the negative and positive predictive values for PCR and IgM test were evaluated. For PCR test, the sensitivity was 0.431, specificity was 0.985, and the negative and positive predictive values were 0.506 and 0.980, respectively. For IgM test, the sensitivity was 0.151, specificity was 0.970, and the negative and positive predictive values were 0.403 and 0.895, respectively.

For cases with clinical diagnosis, the PCR positivity values were 0.158, 0.630, 0.788, and 0.435 for HSV, VZV, CMV, and *T. gondii*, respectively, whereas the IgM positivity for clinically diagnosed cases were 0.684, 0.444, 0.082, and 0.087 for HSV, VZV, CMV and *T. gondii*, respectively (Table 2).

**Table 2:** Diagnostic parameters of aqueous humor PCR and serum IgM test for clinical diagnosis

	Sensitivity	Specificity	Negative predictive value	Positive predictive value
**2a**				
**Aqueous PCR**	0.431	0.985	0.506	0.980
**Serum IgM**	0.151	0.970	0.403	0.895
**2b**				
**Clinical diagnosis**	**HSV**	**VZV**	**CMV**	***T. gondii***
**PCR positivity**	0.158	0.630	0.788	0.435
**IgM positivity**	0.684	0.444	0.082	0.087

The sensitivity, specificity, negative and positive predictive values for PCR and IgM test were evaluated. The diagnostic positivity with PCR and IgM was calculated for each clinical diagnosis. *PCR* polymerase chain reaction, *IgM* immunoglobulin M*, T. gondii Toxoplasma gondii, HSV* Herpes simplex virus, *VZV* Varicella-zoster virus, *CMV* Cytomegalovirus

### PCR vs. IgM serologic tests for detecting infectious agents in anterior uveitis

Among the 34 patients with anterior uveitis, PCR results were positive in 10 (29%) patients and IgM serologic test results were positive in 2 (6%) patients. Pathogens detected by PCR were 7 cases of CMV, 2 of HSV, and 1 of VZV. Pathogens detected by IgM serologic tests were 1 case each of HSV and VZV (Table 1).

### PCR vs. IgM serologic tests for detecting infectious agents in intermediate uveitis

Among the 10 patients with intermediate uveitis, the diagnostic positivity of PCR and IgM serologic tests was 0% (0) and 10% (1 patient), respectively. Although PCR detected no pathogen, IgM serologic tests detected one case of HSV (Table 1).

### PCR vs. IgM serologic tests for detecting infectious agents in posterior uveitis

Among the 22 patients with posterior uveitis, PCR results were positive in 1 (5%) patient, and IgM serologic test results were positive in 0 (0%) patients. PCR identified one case of CMV, whereas IgM serologic tests identified none (Table 1).

### PCR vs. IgM serologic tests for detecting infectious agents in panuveitis

Among the 292 patients diagnosed with panuveitis, the diagnostic positivity of PCR and IgM serologic tests was 30% (88 patients) and 12% (35 patients), respectively. PCR identified 59 cases of CMV, 16 of VZV, 10 of *T. gondii*, 2 of EBV, and 1 of HSV. IgM serologic tests identified 11 cases of HSV, 11 of VZV, 7 of CMV, 4 of EBV, and 2 of *T. gondii* (Table 1).

### Aqueous humor sampling-related complications

No significant adverse events associated with aqueous humor sampling were observed during the follow-up period. No significant cataract progression occurred, and no cases of intraocular hypertony or hypotony were observed. No statistically significant differences in intraocular pressure were observed between the groups during the follow-up period (data not shown). Moreover, no patient reported adverse systemic events during the follow-up period.

## Discussion

This study reports an overall diagnostic positivity of 28% for PCR compared with 11% for IgM serologic tests to identify uveitis-associated infectious agents. A gold-standard diagnostic tool to detect infectious uveitis is lacking despite numerous studies. Clinical diagnosis based on medical history and ocular examination remains the widely accepted methodology; however, few supportive diagnostic tools exist that help to confirm the primary clinical diagnosis.

Aqueous humor PCR has been proposed as a possible diagnostic tool in several studies [7, 10, 12, 17, 18]. However, the usefulness of PCR in the diagnosis of infectious uveitis remains controversial. A retrospective study reported a 10% diagnostic positivity of aqueous humor PCR for diagnosing anterior uveitis [17], whereas another study has reported a diagnostic positivity of 30% [12]. These diverging results can be attributed to different study designs and the type of uveitis [17, 19]. The geographical region and patient population can also affect study results based on epidemiologic variabilities in the spread of viruses. Furthermore, some institutions perform routine PCR for all patients with uveitis, while others decide on a case-to-case scenario based on clinical suspicion [17, 19].

**Fig. 1:**
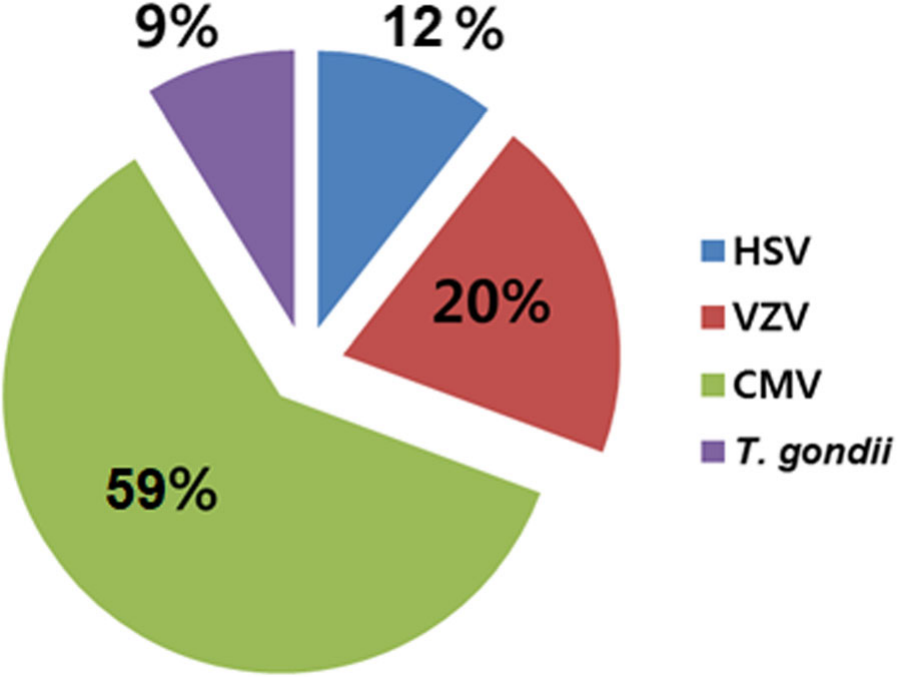
Pathogen type in infectious uveitis. The prevalence of pathogen type in infectious uveitis (*N* = 116) was confirmed based on clinical diagnosis and aqueous humor polymerase chain reaction or immunoglobin M serologic tests. HSV, herpes simplex virus; VZV, varicella-zoster virus; CMV, cytomegalovirus; *T. gondii*, *Toxoplasma gondii*

In South Korea, there are some reports on the clinical patterns of uveitis but none on the prevalence of pathogens identified in infectious uveitis cases. In this study, the pathogens detected using either PCR or serologic tests were CMV in 69 (59%) patients, VZV in 23 (20%), HSV in 14 (12%), and *T. gondii* in 10 (9%) (Fig. 1). CMV and VZV were the causative pathogens in the majority (79%) of infectious uveitis cases. These results differ largely with those reported in other studies on non-Asian Caucasian patients in which HSV, VZV, and *T. gondii* were the most commonly identified pathogens [20–22]. Some geographically proximal countries to South Korea, such as India, Africa, and Japan, have reported HSV, *T. gondii*, and *Mycobacterium tuberculosis* as the most commonly detected pathogens in patients with uveitis [22–26].

In this study, EBV was detected by both PCR and IgM serologic tests in two patients with panuveitis and only by IgM serologic tests in two patients with acute retinal necrosis (ARN), one of whom had a co-infection with CMV, and CMV caused the primary infection. In two patients with clinically diagnosed VZV-uveitis, laboratory test results revealed dual infection (EBV + VZV). EBV is considered to infect the ocular pigment epithelial cells [27], but some studies consider EBV as a secondary factor in ocular inflammation rather than as the primary infectious cause [12, 28]. A few studies have reported that EBV infection might result in ARN; however, this association remains controversial [29]. Therefore, more evidence is required to clarify the role of EBV in uveitis.

For IgM testing, there are two aspects that need to be considered. First, despite low sensitivity, the specificity of IgM test is relatively high, with a low false positive value (sensitivity was 0.151, specificity was 0.970, and the negative and positive predictive values were 0.403 and 0.895, respectively, in our study). Second, as there are endemic areas of viral infection, patients might be broadly positive for IgG; this generalized IgG positivity might not provide any evidence for diagnosis for acute infectious uveitis. For example, more than 90% of Koreans are positive for anti-CMV IgG [30]. Overall, we think it is clinically significant to compare the diagnostic value of IgM and PCR, as both diagnostic tests have their own distinct role as an adjuvant diagnostic tool in infectious uveitis.

In the real world, clinical features are always important in establishing prompt diagnosis for appropriate management of infectious uveitis, as early detection of the causative pathogen and appropriate antimicrobial therapy are critical in preventing visual impairment from infectious uveitis. Concurrently, detection of viral DNA from either aqueous humor or vitreous is necessary for final confirmation of clinical diagnosis. In cases where the PCR results were negative, but infectious etiology of uveitis is highly suspected, we tried to obtain more diagnostic clue from the serologic testing.

This study had some limitations. First, this was a single-center retrospective study and was limited to a specific patient population that visited a tertiary, referral-based university hospital located in the capital of South Korea. These factors could have introduced potential bias in the study group. To indicate a more representative Korean population for studying infectious epidemiology in uveitis, we recommend additional multicenter large-sample studies. Second, PCR was performed only when an infectious etiology was suspected based on clinical findings of uveitis. This could have underestimated the diagnostic value of PCR. Third, PCR was not performed for all etiologic agents in a patient because of the small volume of aqueous humor samples, laboratory limitations, and financial burden on the patient. Only the most probable viral markers were tested, and this might have further underestimated the diagnostic value of PCR. Finally, a number of patients had unclear diagnosis because of negative PCR and serologic test results, and in whom ophthalmic findings were used as the gold-standard method for clinical diagnosis (Table 3).

**Table 3:** Clinical diagnoses for those patients whose PCR and serology testing were both negative

Clinical diagnosis	Anatomical classification of uveitis				
	Anterior uveitis (*N* = 24)	Intermediate uveitis (*N* = 9),	Posterior uveitis (*N* = 21)	Panuveitis (*N* = 184).	Total (*N* = 238)
Herpetic keratitis	2	0	0	0	2
Herpes uveitis	0	0	0	3	3
Zoster ophthalmicus	2	0	0	2	4
Endotheliitis	5	0	0	0	5
CMV retinitis	0	0	0	16	16
T. gondii	0	0	3	10	13
Toxocariasis	0	0	2	5	7
Pseudomonas	0	0	0	1	1
Fungal	0	0	0	3	3
Syphilis	0	0	0	2	2
Tuberculosis	0	0	0	2	2
Endophthalmitis	0	0	0	24	24
Lymphoma	0	0	0	4	4
Acute retinal necrosis	0	0	0	19	19
Unclassified	15	9	16	93	133

Results are presented as *N*; *PCR* polymerase chain reaction, *HSV* herpes simplex virus, *VZV* varicella-zoster virus, *CMV* cytomegalovirus, *T. gondii* Toxoplasma gondii

## Conclusions

Aqueous humor PCR demonstrates a 28% diagnostic positivity in patients with suspected infectious uveitis. It can be a valuable diagnostic tool for confirming the causative pathogen. In particular, aqueous humor PCR demonstrated good diagnostic value for identifying the infectious etiology of anterior uveitis and panuveitis as compared with that for intermediate and posterior uveitis (*P* = 0.012).

## Data Availability

The datasets used and/or analyzed during the current study are available from the corresponding author on reasonable request.

## Abbreviations

ARN: Acute retinal necrosis
CMV: Cytomegalovirus
EBV: Epstein-Barr Virus
ELISA: Enzyme-linked immunosorbent assay
HSV: Herpes simplex virus
GWC: Goldmann-Witmer coefficient
PCR: Polymerase chain reaction
VZV: Varicella-zoster virus
